# Tree-ring stable isotopes and growth trajectories reveal early warning signals of drought-induced Scots pine mortality

**DOI:** 10.1093/treephys/tpag059

**Published:** 2026-04-30

**Authors:** Otmar Urban, Claudia Hartl, Christian Zang, Natálie Pernicová, Josef Čáslavský, Kerstin Treydte, Lea Schneider, Miroslav Trnka, Jan Esper, Ulf Büntgen

**Affiliations:** Global Change Research Institute of the Czech Academy of Sciences, Bělidla 986/4a, 60300 Brno, Czech Republic; Nature Rings – Environmental Research and Education, 55118 Mainz, Germany; Panel on Planetary Thinking, Justus-Liebig-University, Liebigstrasse 35, 35392 Gießen, Germany; Department of Forestry, Weihenstephan-Triesdorf University of Applied Sciences, Hans-Carl-von-Carlowitz-Platz 3, 85354 Freising, Germany; Institute of Ecology and Landscape, Weihenstephan-Triesdorf University of Applied Sciences, Am Hofgarten 1, 85354 Freising, Germany; Global Change Research Institute of the Czech Academy of Sciences, Bělidla 986/4a, 60300 Brno, Czech Republic; Global Change Research Institute of the Czech Academy of Sciences, Bělidla 986/4a, 60300 Brno, Czech Republic; Swiss Federal Institute for Forest, Snow and Landscape Research WSL, Zürcherstrasse 111, 8903 Birmensdorf, Switzerland; Department of Geography, Justus-Liebig-University, Senckenbergstrasse 1, 35390 Gießen, Germany; Center for International Development and Environmental Research, Justus-Liebig-University, Senckenbergstrasse 3, 35390 Gießen, Germany; Global Change Research Institute of the Czech Academy of Sciences, Bělidla 986/4a, 60300 Brno, Czech Republic; Global Change Research Institute of the Czech Academy of Sciences, Bělidla 986/4a, 60300 Brno, Czech Republic; Department of Geography, Johannes Gutenberg University, Becherweg 21, 55099 Mainz, Germany; Global Change Research Institute of the Czech Academy of Sciences, Bělidla 986/4a, 60300 Brno, Czech Republic; Department of Geography, University of Cambridge, Downing Place, Cambridge, CB2 3EN, UK; Department of Geography, Faculty of Science, Masaryk University, Kotlářská 267/2, 61137 Brno, Czech Republic

**Keywords:** basal area increment, crown conditions, drought and heat stress, forest resilience, tree mortality, tree vitality

## Abstract

Summer droughts have affected tree growth in central Europe since at least the 1940s, yet the physiological mechanisms behind why some trees die whilst others survive remain poorly understood. Here, we present absolutely dated and annually resolved tree-ring width, carbon (δ^13^C) and oxygen (δ^18^O) stable isotope chronologies from 18 Scots pines (*Pinus sylvestris* L.) growing near the species’ climatic and edaphic limit in one of Germany’s driest regions (Rhine Hesse, near Mainz). Spanning the period 1930–2019, sampled trees were assigned to three post-2018 vitality classes based on crown transparency: vigorous, intermediate and poor vigour (including dead individuals). We assessed long-term growth and isotopic trajectories in relation to climate variables, with a focus on pan-European summer drought extremes in 1947, 1976, 2003 and 2018. We found that trees with consistently higher growth rates and elevated long-term δ^13^C values were more susceptible to dieback, whereas surviving trees maintained lower δ^13^C values. This pattern suggests differences in long-term water-use strategies and/or drought exposure, whilst also partly reflecting canopy position and light-driven assimilation. In contrast, δ^18^O values sharply increased during drought events, especially in poor-vigour trees, indicating greater reliance on shallow, evaporatively enriched water sources and heightened hydraulic strain under extreme drought. These isotopic trajectories differentiated vitality classes well before visible canopy decline. Our findings indicate that the 2018 drought was not the sole trigger, but rather a tipping point in a long-term dieback process driven by repeated droughts and heatwaves. Tree-ring stable isotopes, particularly when interpreted alongside growth trajectories, provide valuable early warning signals of physiological stress and drought vulnerability. Since trees with conservative growth strategies were more resilient, long-term physiological stability, rather than maximum productivity, may enhance forest resilience under an increasingly warm, dry and variable future climate.

## Introduction

Global climate change poses a major threat to forest ecosystems worldwide, affecting their functioning, productivity and resilience (Boisvenue and Running 2006, Trumbore et al. 2015). Although forest decline has been a concern for over half a century, its underlying causes have shifted over time (Tew et al. 2024). Whilst air pollution was considered the primary driver from the 1960s to the 1980s (Ulrich and Pankrath 1983, Schütt and Cowling 1985, Elling et al. 2009), climate change and its cascading effects have become the dominant forces in recent decades. These changes have increased the frequency and severity of forest disturbance events, including drought-induced tree mortality, windthrow and outbreaks of insects and pathogens (Seidl et al. 2014, Adams et al. 2017, Blumenstein et al. 2021).

Amongst these disturbances, summer droughts have emerged as a key driver of forest dieback in Europe (Senf et al. 2020). The unprecedented drought of 2018 (Peters et al. 2020, Büntgen et al. 2021) pushed disturbance regimes beyond their historical variability (Senf and Seidl 2021), triggering widespread physiological stress and causing substantial tree damage and mortality across central Europe (Brun et al. 2020, Fu et al. 2020, Büntgen et al. 2021). However, dieback often occurs selectively, affecting individual trees rather than entire stands (Schuldt et al. 2020). Although the term ‘Waldsterben 2.0’ (i.e., ‘forest dieback 2.0’) has been coined, it inadequately describes the current phenomenon, which is more spatially patchy and species-specific.

Drought, particularly when combined with elevated temperatures, can trigger multiple physiological constraints. Stomatal closure, a common response to water deficit, reduces water loss through transpiration but also limits CO_2_ uptake, potentially leading to reduced growth and carbon starvation (McDowell et al. 2008, Muller et al. 2011, 2022, Adams et al. 2017). Simultaneously, declining water potentials and xylem embolism impair hydraulic conductivity, limiting water transport from roots to leaves and thereby increasing the risk of hydraulic failure, leaf desiccation and ultimate tree mortality (Meinzer and McCulloh 2013, Adams et al. 2017). These stress responses are often recorded in tree rings as reductions in radial growth and shifts in the composition of stable isotopes.

Under drought conditions, elevated δ^13^C values in tree rings typically reflect reduced intercellular CO_2_ concentrations in leaves and increased intrinsic water-use efficiency (iWUE; the ratio of carbon assimilation to stomatal conductance), due to stomatal closure during periods of water limitation (Cernusak 2020, Shestakova and Martínez-Sancho 2021). In parallel, δ^18^O enrichment in tree-ring cellulose primarily reflects evaporative enrichment of leaf water under hot and dry atmospheric conditions (Barbour 2007, Treydte et al. 2024), as the isotopic signal is transferred from leaves to wood via phloem-transported carbohydrates. Importantly, δ^18^O values can also reflect the isotopic composition of source water taken up by the roots, which typically varies with soil depth—surface water tends to be more enriched due to evaporation, whilst deeper water sources retain lower δ^18^O values (Gazis and Feng 2004, Gessler et al. 2014, Treydte et al. 2014). Thus, stable isotope ratios in tree rings reflect an integrated signal of plant physiological processes and environmental conditions, including stomatal regulation, evaporative demand, rooting depth and source water dynamics.

When heat and drought coincide, physiological stress is amplified through increased evaporative water loss, photoinhibition and respiration, resulting in intensified physiological strain and delayed recovery (Gessler et al. 2018, Cherubini et al. 2021). Prolonged or repeated drought may also increase susceptibility to biotic stressors, further raising the risk of mortality (Suzuki et al. 2014, Teshome et al. 2020).

**Figure 1:**
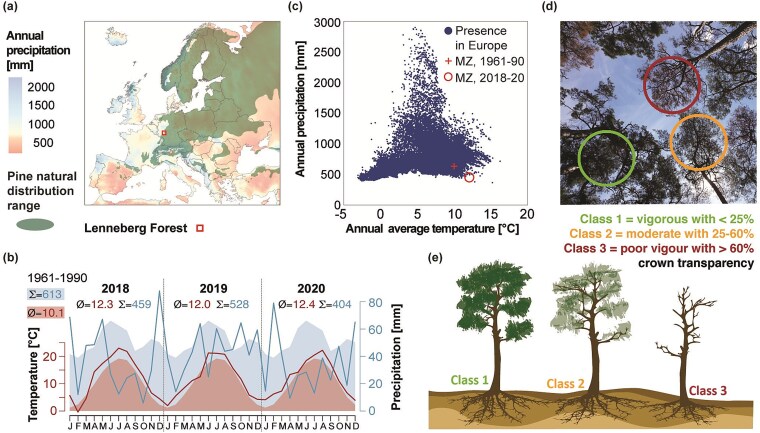
(a) Natural distribution range of Scots pine and the location of the ‘Lenneberg Forest’ in Mainz, Germany. (b) Climate diagram for the 1961–1990 period (area signature) superimposed with the climatic conditions of the subsequent drought events 2018, 2019 and 2020 (line signatures) in Mainz (station ID 3137, German Weather Service, DWD). (c) Climate envelope of Scots pine with its occurrences in Europe (dots, reproduced from Houston Durrant et al. 2016). The cross represents the climatic conditions of Mainz (= MZ) in the 1961–1990 period, and the circle represents the conditions during the years 2018–2020. (d) Tree vigour at the study site with different crown conditions/transparency and (e) classification of crown conditions representing tree vigour. Transparency classes denote canopy condition assessed in 2019; partial defoliation near 60% can be acclimatory in some individuals.

Scots pine (*Pinus sylvestris* L.) is one of the most widespread and economically important conifers in Europe (Figure 1a) (Brus et al. 2012, Houston Durrant et al. 2016). Although naturally adapted to a broad range of environments, its distribution has been expanded through afforestation to marginal sites (Houston Durrant et al. 2016), such as Rhine Hesse—one of Germany’s driest regions—where increasing drought sensitivity has been reported (Haberstroh et al. 2022). Scots pine has also become more vulnerable to secondary stressors, including pathogens such as *Diplodia sapinea* (Brodde et al. 2019, Blumenstein et al. 2021).

Even within a single stand, tree responses to drought vary: some individuals die, whilst others survive or decline more gradually (Figure 1; Bose et al. 2024). The causes of this variability remain insufficiently understood, and explanations for partial dieback are still lacking. Whilst many studies rely on growth-based indicators such as radial increment and canopy transparency to assess tree vitality (Dobbertin 2005), few integrate stable isotope analyses. Tree rings can provide long-term records of past physiological functioning and environmental impacts (Fritts 1976), allowing retrospective assessment of growth disturbances and recovery dynamics (Hartl-Meier et al. 2017, Hartl et al. 2019, 2022, Harr et al. 2021).

Isotopic ratios (δ^13^C, δ^18^O) offer additional insights into key physiological processes—such as stomatal regulation of gas exchange, iWUE, evaporative water loss and whole-tree water balance—that underlie drought coping strategies at both the species and individual tree level (Hartl-Meier et al. 2015, Cherubini et al. 2021, Rybníček et al. 2021, Shestakova and Martínez-Sancho 2021). Although these metrics have been widely used to detect drought stress (Gessler et al. 2018, Cherubini et al. 2021, Pernicová et al. 2024), they have rarely been applied to explain intraspecific variation in tree vitality and survival. Comparing the isotopic and growth responses of surviving and dying trees may thus help identify early warning signals of mortality risk (Camarero et al. 2015, Cailleret et al. 2017, DeSoto et al. 2020, Camarero 2021, Bose et al. 2024).

To address this gap, we combine here annually resolved and absolutely dated tree-ring width and dual-isotope (δ^13^C, δ^18^O) chronologies from Scots pine trees of different vitality classes. We aim to assess whether long-term physiological signals preserved in tree rings can explain differential drought responses amongst coexisting individuals. Specifically, we test the hypothesis that trees with consistently higher growth rates and elevated δ^13^C values are more vulnerable to drought-induced dieback, and that δ^18^O signals during drought years differ systematically amongst vitality classes. Our goal is to determine whether these combined indicators can be used as early warning proxies for drought vulnerability, detectable prior to visible decline.

## Materials and methods

### Investigation area

The study site is located in the ‘Lenneberg Forest’ near Mainz, western Germany (50°00′44.0″N, 8°11′23.7″E; ~ 150 m a.s.l.). The surrounding Rhine Hesse region is amongst the warmest and driest areas in central Europe (Figure 1a and c). With an annual precipitation of ~ 600 mm and a mean annual temperature of ~ 10 °C (climate data for the 1961–1990 reference period, DWD station ID 3137; Figure 1b), the region is also known for its suitability for viticulture. The stand under investigation is part of a permanent forest-condition monitoring plot covering 19.1 ha and comprising 1941 trees (~102 stems ha^−1^). Reported tree mortality was 5.2% for 2018–2021 and 3.4% for 2021–2022 (MUEEF 2022).

The sampling site itself is a mixed plantation established in the 1870s on flat terrain with minimal micro-site variation, dominated by Scots pines (*Pinus sylvestris* L.) and European beech (*Fagus sylvatica* L.). Forest stands in the area grow on aeolian sands and scattered dune deposits with low water-holding capacity, which likely exacerbates drought stress by limiting plant-available water during prolonged dry periods. The current structure reflects both historical silvicultural interventions (e.g., thinning) and density-dependent self-thinning over the past century. Long-term competitive interactions have thus likely influenced early growth trajectories, rooting depth and crown stratification. Although precise historical mortality rates since establishment are unavailable, the even-aged structure and declining stem density suggest significant past mortality. To further characterize recent drought impacts at the canopy scale, annual summer (June–August) satellite composites for a 2-ha region of interest centred on the sampling location revealed pronounced drought-year anomalies (Figure S1 available as Supplementary Data at *Tree Physiology* Online). Landsat NDVI (Normalized Difference Vegetation Index) decreased by 8.2% in 2003 relative to 2000–2002 and by 7.0% in 2018 relative to 2015–2017. Similar declines (7.5% in 2003, 6.4% in 2018) were observed in Sentinel-2 NDRE (Normalized Difference Red Edge) signals, indicating reduced canopy chlorophyll content and vitality (see Figure S1 available as Supplementary Data at *Tree Physiology* Online for detailed methodology).

### Study design and sampling

To capture a representative range of canopy conditions and tree vitality (Figure 1d and e), we selected 18 Scots pine trees spanning a gradient from vigorous to severely declining individual, including recently dead trees. For each selected tree, we recorded geographic coordinates, diameter at breast height (DBH), and visually assessed crown transparency in 5% intervals following the German Forest Administration standard protocol (Wellbrock et al. 2018). Crown transparency was used as a proxy for current canopy vitality and photosynthetic potential, consistent with national forest health monitoring approaches (e.g., Seidling et al. 2012). Based on this measure, trees were grouped into three classes: Class 1—low crown transparency (<25%), interpreted as vigorous canopy condition; Class 2—moderate transparency (25–60%), indicating intermediate canopy condition; and Class 3—high transparency (>60%), corresponding to poor canopy condition and including recently dead trees. We refer to these groups as vitality classes for brevity and consistency with common usage in forest monitoring, though we acknowledge that crown transparency alone does not fully capture functional vigour or future survival potential (Tallieu et al. 2020). Partial defoliation near the 60% threshold, for example, may reflect acclimatory canopy restructuring rather than imminent mortality (cf. German Forest Health Survey protocol). Each class comprised six individuals.

Each tree was cored twice at breast height in opposite directions in December 2019 using a 0.5 mm increment borer. Cores were cut using a core microtome (Gärtner and Nievergelt 2010) to obtain clean transverse surfaces. Tree-ring widths were measured at 0.01 mm precision using a LINTAB measuring table and TSAP-Win software (Rinntech, Heidelberg, Germany). Visual cross-dating was verified statistically with the COFECHA programme (Holmes 1983).

One core per tree was used for stable isotopic analysis at the Global Change Research Institute (CzechGlobe), Czech Republic. Annual rings from 1930 to 2019, including both early and latewood, were separated with a scalpel. In this study, holocellulose was extracted from individual rings using a modified Jayme-Wise method, in which samples were washed in 5% NaOH and subsequently treated with 7% NaClO_2_ at pH 4–5 (Gaudinski et al. 2005). Tree rings were processed separately for each tree and year, i.e., without pooling.

Approximately 1.0 mg of dried, homogenized cellulose was combusted to CO_2_ (for δ^13^C at 960 °C) or pyrolyzed to CO (for δ^18^O at 1450 °C) using a varioPYRO cube elemental analyzer (Elementar Analysensysteme, Langenselbold, Germany). Isotope ratios were determined using an ISOPRIME100 mass spectrometer (Isoprime, Manchester, UK) operating in continuous-flow mode. The δ^13^C (^13^C/^12^C) and δ^18^O (^18^O/^16^O) values are expressed in per mil (‰) relative to Vienna Pee Dee Belemnite (VPDB) and Vienna Standard Mean Ocean Water (VSMOW), respectively, using the formula δ = (*R*_sample_/*R*_standard_ − 1) × 1000, where *R* is the ratio of heavy to light isotopes. Analytical precision, based on the standard deviation of five replicate measurements of a homogenized cellulose sample, was better than 0.04‰ for δ^13^C and 0.19‰ for δ^18^O. The δ^13^C values were corrected for the Suess effect (i.e., atmospheric δ^13^C decline due to fossil fuel emissions) following Belmecheri and Lavergne (2020). For further analytical details, see Urban et al. (2021).

### Statistical analysis

To assess the quality and coherence of the tree-ring width and isotope chronologies, we calculated standard dendrochronological statistics for each vitality class and for the full dataset. These included the first-order autocorrelation (Lag-1), which indicates the influence of previous year’s growth or isotopic signal; the mean inter-series correlation (Rbar), reflecting the common signal amongst series; and the expressed population signal (EPS), which estimates the statistical reliability of the sample to represent a theoretical population.

To remove age-related growth trends, basal area increment (BAI) was calculated from tree-ring width and DBH data for each tree (Biondi and Qeadan 2008). Relationships between tree-ring variables (BAI, δ^13^C and δ^18^O) and environmental factors were assessed using bootstrapped correlation analyses. Climate variables included monthly means of temperature (Temp_mean_), maximum temperature (Temp_max_) and precipitation (Prec), derived from the E-OBS v23.1e gridded dataset (0.25° resolution; Cornes et al. 2018). Additional variables included potential evapotranspiration (Pet) and the 1-month standardized precipitation-evapotranspiration index (Spei1), calculated according to Vicente-Serrano et al. (2010). Soil moisture index (Smi) data at 0.25 m and 1.8 m depths were retrieved from the German Drought Monitor Centre (Samaniego et al. 2013).

Correlation analyses were performed for each calendar month from June of the year preceding tree-ring formation to December of the current growth year. This was applied to both climate data (1930–2019) and soil moisture data (1952–2019). In addition, we tested seasonal (April–September) means and sums for the same variables. Monthly correlation results are presented in Figures S2–S4 available as Supplementary Data at *Tree Physiology* Online, whilst seasonal correlations are presented below in the Results. Dendrochronological statistics and climate–growth correlation analyses were conducted in R v4.2.0 (R Core Team 2022) using packages *dplR* (Bunn et al. 2021), *SPEI* (Beguería and Vicente-Serrano 2017), *pastecs* (Grosjean and Ibanez 2018) and *treeclim* (Zang and Biondi 2015).

To assess differences in BAI, δ^13^C and δ^18^O amongst tree vigour classes (Class 1–3) across the 1930–2019 period, we applied a combination of linear mixed-effects models (LMMs) and non-parametric tests. Each class comprised six individual trees (T1–T6). Because all tree-ring variables were annually resolved, the dataset included repeated measurements for each tree across the 90-year time series.

To evaluate overall differences amongst classes and to examine long-term temporal trends, two LMMs were fitted. The first model tested for class-level differences in mean BAI, δ^13^C and δ^18^O values across the entire study period, with tree identity as a random intercept. The second model included vitality class, calendar year and their interaction as fixed effects, again with tree identity as a random effect, allowing us to assess class-specific temporal trends and their statistically significant differences. Models were fitted using restricted maximum likelihood (REML).

In addition to LMMs, we conducted year-by-year pairwise comparisons using the non-parametric Mann–Whitney U test to identify specific years with significant differences in BAI, δ^13^C or δ^18^O between vitality classes. Comparisons were performed separately for each year and each pair of classes (Class 1 vs 2, Class 1 vs 3 and Class 2 vs 3). Whilst unadjusted *P*-values are reported for transparency, Bonferroni corrections were applied to control for multiple testing. Statistical significance was evaluated at the α = 0.05 level. Linear mixed-effects models and non-parametric tests were conducted in Python using the *pandas*, *statsmodels* and *scipy* libraries.

To evaluate responses to major pan-European drought events (1947, 1976, 2003 and 2018), BAI changes (percent deviation from baseline) and isotopic anomalies were assessed. Anomalies were expressed as scaled deviations relative to the 5-year mean preceding each drought event, following Hartl-Meier et al. (2014, 2015) and Hartl et al. (2019).

## Results

### Characteristics of tree-ring data and vigour classes

**Figure 2:**
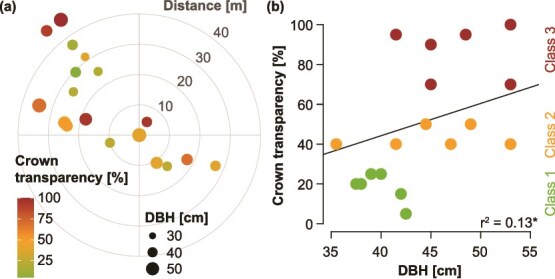
(a) Spatial distribution of the sampled trees at the study site, showing variation in diameter at breast height (DBH) and crown transparency. (b) Relationship between crown transparency (%) and DBH (cm) based on individual tree data. The black line represents the linear regression model (^*^ indicates *P* < 0.1).

The sampled Scots pine trees had a cambial age of ~137 years at breast height and were even-aged, consistent with their plantation origin. Each vitality class consisted of six trees randomly distributed across the site, with no evident spatial clustering (Figure 2a). A weak positive relationship was observed between tree diameter at breast height (DBH) and crown transparency, with larger DBH associated with greater crown transparency, although the explained variance was low (R^2^ = 0.13, *P* < 0.1; Figure 2b).

Tree-ring parameters showed high internal coherence, with mean inter-series correlation (Rbar) values of 0.62 for tree-ring width, 0.61 for δ^13^C and 0.47 for δ^18^O (Table 1). First-order autocorrelation (Lag-1) was highest for tree-ring width (0.80), followed by δ^13^C (0.33) and δ^18^O (0.18). These values varied slightly amongst vitality classes (Table 1).

**Table 1:** Characteristics of the raw TRW, δ^13^C and δ^18^O chronologies for all trees and vitality classes. SD = standard deviation, Lag-1 = first-order autocorrelation, Rbar = mean inter-series correlation, EPS = expressed population signal, indicating the strength of the common signal and adequacy of sample replication. All values calculated for the 1930–2019 period.

		Mean	Median	SD	Lag-1	Rbar	EPS
**TRW**	All	1.37	1.18	0.85	0.79	0.62	0.96
	Class 1	1.30	1.05	0.96	0.80	0.59	0.89
	Class 2	1.35	1.12	0.85	0.82	0.72	0.94
	Class 3	1.47	1.37	0.74	0.75	0.58	0.88
**δ** ^**13**^ **C**	All	−22.20	−22.20	0.80	0.33	0.61	0.96
	Class 1	−22.35	−22.36	0.76	0.31	0.51	0.85
	Class 2	−22.44	−22.45	0.78	0.33	0.65	0.91
	Class 3	−21.81	−21.79	0.86	0.35	0.65	0.92
**δ** ^**18**^ **O**	All	32.06	32.01	0.99	0.18	0.47	0.94
	Class 1	31.72	31.68	1.09	0.17	0.40	0.78
	Class 2	32.58	32.55	0.86	0.13	0.58	0.89
	Class 3	31.89	31.79	1.03	0.22	0.49	0.85

### Basal area increment

**Figure 3:**
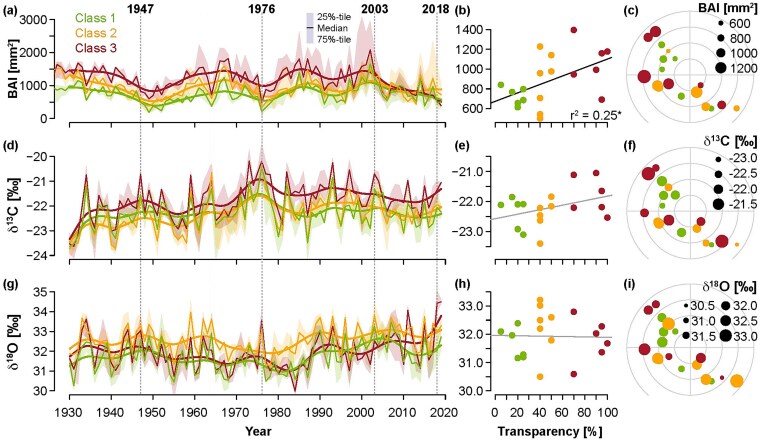
Tree-ring parameters and their relationships to crown condition and spatial distribution. (a, d, g) Time series of BAI, δ^13^C and δ^18^O for all trees. Thin lines show median values for each vigour class, thick lines represent 15-year smoothing, and shaded areas indicate 25th–75th percentiles. (b, e, h) Relationships between tree-level median BAI, δ^13^C and δ^18^O values and crown transparency. Solid black regression lines are statistically significant (*P* < 0.05), whilst grey lines are non-significant. (c, f, i) Spatial distribution of tree-level median BAI, δ^13^C and δ^18^O across the study site. Circle size indicates the magnitude of each parameter.

The BAI time series revealed a clear relationship between long-term growth and current tree vitality (Figure 3a). Trees in Class 3 (poor vigour) exhibited consistently higher BAI values in the early part of the record (Table S1 available as Supplementary Data at *Tree Physiology* Online), but their growth declined more sharply over time. In contrast, Class 1 (vigorous) trees showed lower initial growth but maintained more stable rates in recent decades.

Linear mixed-effects modelling (LMM) confirmed these trends. In Model 2, which included the Class × Year interaction, BAI was significantly higher in Class 3 than Class 1 (*P* = 0.004), but this difference declined over time due to a significant negative Class 3 × Year interaction (*P* = 0.032; Table S2 available as Supplementary Data at *Tree Physiology* Online). This supports the interpretation that rapid early growth may predispose trees to long-term decline under increasing drought stress.

Class 1 trees showed a marginally positive BAI trend over time (*P* = 0.069), whilst no significant differences were detected between Class 2 and Class 1. Additionally, median BAI values explained 25% of the variation in crown transparency (*P* < 0.1; Figure 3b), reinforcing the link between historical growth patterns and current tree condition. No spatial pattern was observed for median BAI values (Figure 3c).

### Carbon isotope composition (**δ**^13^C)

No clear visual differences were evident in δ^13^C chronologies across vitality classes (Figure 3d and e). However, LMM analysis revealed significant differences in both intercept and slope of δ^13^C trajectories (Model 2; Table S2 available as Supplementary Data at *Tree Physiology* Online). Trees in Class 2 and Class 3 had significantly lower initial δ^13^C values than Class 1 (*P* = 0.001 and *P* = 0.004, respectively), but exhibited steeper increasing trends over time (Class 2 × Year: *P* = 0.001; Class 3 × Year: *P* = 0.004). These divergent trajectories suggest that less vigorous trees experienced progressively greater physiological stress and increasingly conservative stomatal regulation.

Despite these temporal trends, average δ^13^C values did not differ significantly across classes when considered over the entire time period (Class 3 vs Class 1: *P* = 0.089). Year-by-year Mann–Whitney U tests identified several isolated years with significant differences between classes, but no consistent pattern emerged (Table S1 available as Supplementary Data at *Tree Physiology* Online).

No spatial autocorrelation was detected in δ^13^C median values across the plot (Figure 3f), supporting the interpretation that observed differences reflect physiological history rather than local microsite effects.

### Oxygen isotope composition (**δ**^18^O)

Similarly, δ^18^O values showed no consistent visual differences across vigour classes (Figure 3g and h). However, LMM analysis revealed a modest but significant increase in δ^18^O over time across all classes (*P* = 0.028), with no significant class-specific trends or intercept differences (Table S2 available as Supplementary Data at *Tree Physiology* Online). Class 2 trees showed slightly higher overall δ^18^O values than Class 1 (*P* = 0.010), whilst Class 3 did not differ significantly from Class 1. Nonetheless, year-by-year Mann–Whitney U tests detected significant differences (*P* < 0.05) between classes in 29 individual years between 1930 and 2019, albeit without a consistent pattern (Table S1 available as Supplementary Data at *Tree Physiology* Online).

Spatial distributions of median δ^18^O values revealed no pattern related to position within the plot (Figure 3i), suggesting that inter-tree variation is primarily driven by intrinsic physiological or rooting differences rather than soil heterogeneity.

### Relationships between tree-ring data and environmental variables

**Figure 4:**
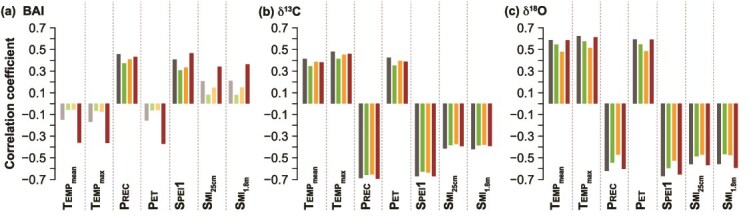
Correlation coefficients between environmental variables during the growing season (April–September) and (a) BAI, (b) δ^13^C and (c) δ^18^O, shown for different vigour classes (colour coded as in the previous figures) and for the overall chronology (grey bars). Only correlations significant at *P* < 0.05 are shown in solid colours; non-significant correlations are displayed as transparent bars.

Monthly correlation analyses revealed that BAI was most responsive to current-year summer conditions, especially in July, irrespective of vigour classes (Figure S2 available as Supplementary Data at *Tree Physiology* Online). Growth was negatively correlated with Temp_mean_, Temp_max_ and Pet, and positively correlated with Prec and Spei1. Amongst the vigour classes, Class 3 consistently showed the strongest correlations. When seasonal (April–September) means or sums were considered, significant correlations with Temp_mean_, Temp_max_ and Pet were found only for Class 3. Shorter seasonal aggregations of environmental conditions yielded lower correlations (Figure 4a).

For the soil moisture index (Smi) at both 0.25 and 1.8 m depths, only Class 3 showed significant correlations, further highlighting the heightened sensitivity of low-vigour trees to reduced water availability (Figure 4a, Figure S2 available as Supplementary Data at *Tree Physiology* Online). No significant correlations with SMI were observed for Classes 1 or 2, nor for the overall BAI chronology.

In contrast, δ^13^C values showed stronger and more consistent correlations with environmental variables compared with BAI (Figure 4b, Figure S3 available as Supplementary Data at *Tree Physiology* Online). The δ^13^C values were positively correlated with Temp_mean_, Temp_max_ and Pet and negatively correlated with Prec, Spei1 and Smi. Class 3 and the overall δ^13^C chronology exhibited the strongest correlations, with particularly high negative correlations with Prec (*r* = −0.69) and Spei1 (*r* = −0.67) for both Class 3 and all trees combined.

δ^18^O values showed the strongest overall correlations with environmental variables amongst all tree-ring parameters (Figure 4c, Figure S4 available as Supplementary Data at *Tree Physiology* Online). The highest correlations were observed for current-year summer conditions, with positive correlations to Temp_mean_, Temp_max_ and Pet and negative correlations to Prec, Spei1 and Smi. The highest correlation coefficients were observed for Temp_max_ (*r* = 0.61 for Class 3, *r* = 0.62 for all trees) and Spei1 (*r* = −0.65 for Class 3, *r* = −0.67 for all trees). As with δ^13^C, Class 3 and the overall δ^18^O chronology consistently showed the highest sensitivity to all environmental variables tested, reinforcing the utility of δ^18^O as a reliable indicator of drought-induced stress.

### Responses to major drought events

**Figure 5:**
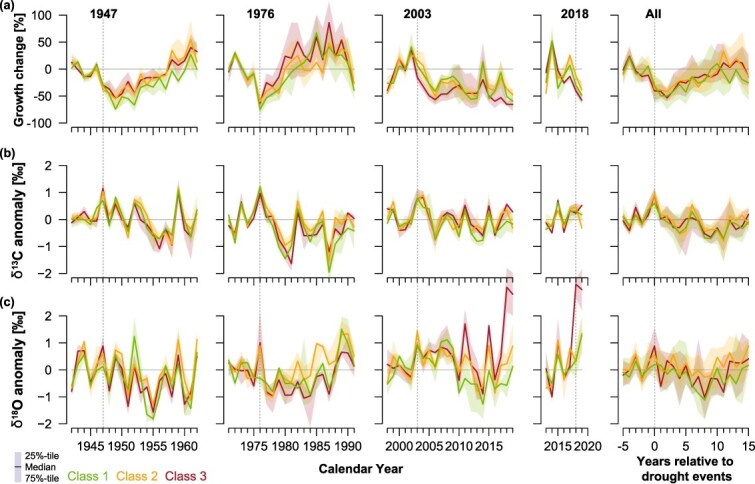
Vigour class-specific response in (a) basal area increment (BAI), (b) δ^13^C and (c) δ^18^O to the drought events of 1947, 1976, 2003 and 2018. Responses are shown as changes relative to the 5 years preceding each drought. The right panels summarize the average responses across all events. Lines represent median values, and shaded areas indicate the interquartile range (25th–75th percentile). Colour coding follows the convention of previous figures.

Tree-ring data revealed distinct patterns of response to major pan-European drought events amongst the vigour classes (Figure 5). During the 1947 and 1976 droughts, growth (represented by BAI) declined similarly across all classes; however, recovery trajectories differed. Notably, Class 1 (vigorous) trees required a considerably longer period to return to pre-drought growth rates. In 2003, only Class 3 (poor vigour) trees exhibited a pronounced growth decline with no recovery. In contrast, Classes 1 and 2 showed delayed reductions and partial recovery in 2014 and 2017, although growth remained lower than in 5 years preceding 2003. The 2018 drought triggered another marked decline in BAI across all classes, with growth dropping below already reduced rates. Whilst many of these differences were visually evident, only the 1976 event yielded statistically significant variation in BAI amongst classes (*P* = 0.047; Table 2), with Class 3 showing higher values than Class 1. These results are consistent with broader year-by-year comparisons presented in Table S1 available as Supplementary Data at *Tree Physiology* Online.

**Table 2:** Mean values ± standard deviations (SD) of annual basal area increment (BAI, mm^2^), carbon isotope composition (δ^13^C, ‰) and oxygen isotope composition (δ^18^O, ‰) in tree rings of Scots pine during major drought years (1947, 1976, 2003 and 2018).

		1947		1976		2003		2018	
		Mean ± SD	*P*	Mean ± SD	*P*	Mean ± SD	*P*	Mean ± SD	*P*
**BAI**	Class 1	490 ± 181		190 ± 128^a^		1193 ± 612		533 ± 309	
	Class 2	530 ± 171		358 ± 156^ab^		1445 ± 777		1031 ± 620	
	Class 3	762 ± 244	0.071	417 ± 191^b^	**0.047**	1556 ± 808	0.690	516 ± 134	0.094
**δ** ^**13**^ **C**	Class 1	−21.58 ± 0.50^a^		−20.53 ± 0.69		−21.49 ± 0.63^ab^		−21.98 ± 0.64	
	Class 2	−21.75 ± 0.30^a^		−20.37 ± 0.15		−21.63 ± 0.66^a^		−21.87 ± 0.42	
	Class 3	−20.75 ± 0.51^b^	**0.003**	−20.21 ± 0.87	0.723	−20.83 ± 0.36^b^	**0.046**	−21.41 ± 0.84	0.475
**δ** ^**18**^ **O**	Class 1	32.56 ± 1.46		32.03 ± 1.55		33.11 ± 1.30		31.78 ± 0.98^a^	
	Class 2	33.33 ± 0.83		33.09 ± 0.59		33.86 ± 0.80		33.11 ± 0.96^ab^	
	Class 3	32.85 ± 1.09	0.523	33.21 ± 1.31	0.332	32.74 ± 1.04	0.202	34.65 ± 0.90^b^	**0.005**

*P*-values indicate differences amongst vigour classes based on one-way ANOVA; bold *P*-values indicate statistically significant differences among vigour classes (*P* < 0.05). For each drought year and parameter, pairwise comparisons between vigour classes were conducted using the non-parametric Mann–Whitney U test (α = 0.05) to identify which class pairs differed significantly. Identical superscript letters (a, b) indicate statistically homogeneous groups (*P* > 0.05). For a complete year-by-year summary of Mann–Whitney U test results across the full chronology (1930–2019), see Table S1 available as Supplementary Data at *Tree Physiology* Online.

The δ^13^C values peaked during drought years for all vigour classes (Figure 5b). In 1947, δ^13^C values were significantly higher in Class 3 than in Classes 1 and 2 (*P* = 0.003), suggesting reduced stomatal conductance and higher iWUE. A similar pattern re-emerged in 2003, when Class 3 again showed significantly elevated δ^13^C values compared with Class 2 (*P* = 0.046; Table 2). Although the δ^13^C response in 2018 was less pronounced, aggregated across all drought years, Class 1 trees consistently showed lower δ^13^C values compared with Class 2 and Class 3 trees, indicating their greater stomatal openness and lower iWUE. These class differences were further supported by significant differences in δ^13^C trajectories over time as revealed by linear mixed-effects modelling (Table S2 available as Supplementary Data at *Tree Physiology* Online).

In contrast, δ^18^O values demonstrated clearer class-based differences during drought years (Figure 5c). During 1947 and 1976, Classes 2 and 3 showed marked δ^18^O increases, whereas Class 1 showed minimal change. A mild δ^18^O rise was observed in Class 1 in 2003, but the strongest divergence occurred in 2018 and continued into 2019, when δ^18^O values surged in Class 3 trees. This difference was statistically significant in 2018 (*P* = 0.005; Table 2), with Class 3 showing substantially higher δ^18^O values than Class 1, likely reflecting enhanced evaporative enrichment and/or greater reliance on evaporatively enriched shallow water, potentially linked to impaired stomatal regulation under extreme drought stress. These findings confirm that δ^18^O is the most responsive indicator for distinguishing drought-related stress amongst vigour classes, as further supported by the complete set of year-to-year comparisons in Table S1 available as Supplementary Data at *Tree Physiology* Online.

## Discussion

We compiled a robust dataset of BAI and tree-ring δ^13^C and δ^18^O records from Scots pine trees growing within a single forest stand that exhibited varying degrees of vitality after the 2018 drought. This dataset allowed us to explore the dieback process and identify physiological signals that precede or predict mortality. The study site—Lenneberg Forest—is located at the climatological margin of Scots pine’s distribution range (Figure 1a and c) and is characterized by sandy soils with low water retention capacity, which exacerbate drought stress. Climate data confirm that the years following 2018 were exceptionally hot and dry, falling outside the historical climate envelope for this species (Figure 1b and c). It is therefore unsurprising that trees are increasingly struggling under such conditions.

### Growth patterns and longevity trade-offs

Long-term growth patterns, based on correlations calculated for the full 1930–2019 period, indicate that tree-ring formation is primarily influenced by July climate conditions, as shown in Figure S2 available as Supplementary Data at *Tree Physiology* Online. Growth declined under hot and dry Julys, with trees of poor vigour—those with reduced leaf area and high crown transparency—showing the strongest sensitivity, particularly to temperature, potential evapotranspiration (PET) and soil moisture index (SMI). We also acknowledge that partial defoliation may represent an acclimatory response in some individuals, especially near the ~ 60% transparency threshold; our ‘poor vigour’ label denotes current condition, not fate. Notably, only these now-poor-vigour trees (Class 3) showed significant correlations with soil moisture (Figure S2 available as Supplementary Data at *Tree Physiology* Online), suggesting shallower root systems and a greater dependence on less stable moisture in upper soil layers. In contrast, trees currently classified as vigorous (Class 1) likely experienced lower drought stress, as indicated by their lower δ^13^C values (Figure 3d–f), which imply lower iWUE and greater stomatal openness (Colangelo et al. 2017, Cernusak 2020, Pernicová et al. 2024). In addition, their systematically lower δ^18^O values after 2003 compared with Classes 2 and 3 support the interpretation that Class 1 trees relied more on isotopically lighter, less evaporatively enriched water, possibly from deeper soil layers. By contrast, the more enriched δ^18^O values in Classes 2 and 3 suggest greater reliance on evaporatively enriched surface water and/or stronger leaf-water enrichment under drought stress (Gazis and Feng 2004, Gessler et al. 2014, Treydte et al. 2014).

Our findings challenge the assumption that high historical growth rates guarantee long-term vitality. Within the current group of trees, those with the highest past BAI values now show the most extensive crown damage. This supports evidence that high biomass productivity may come at the cost of reduced longevity (Büntgen et al. 2019, DeSoto et al. 2020). In contrast, individuals with more conservative long-term growth—those that persisted through repeated droughts—exhibited greater resilience in recent years, reinforcing the idea that ‘slow growers survive’.

We recognize, however, that this interpretation is constrained by survivorship bias (Brienen et al. 2020). Since we only sampled trees still present at the time of sampling and did not include individuals that had died earlier during stand development, we cannot assess whether some of the slowest growers died earlier during stand development. Given the even-aged, plantation origin of the stand, past competition and density-dependent mortality likely shaped early growth trajectories (Pretzsch 2009). Still, the observed trade-off between past growth and current crown condition amongst surviving trees offers valuable insight into drought resilience mechanisms.

Contrasting results from a more xeric Scots pine site in southern Poland (Benisiewicz et al. 2024) showed that trees with higher past growth experienced less drought damage and maintained better physiological performance. These divergent outcomes likely reflect differences in site conditions, rooting depth and drought severity. In extremely dry environments, greater carbon gain and crown development may enhance drought resilience by sustaining root activity and carbohydrate reserves. Conversely, in moderately dry but repeatedly stressed systems like ours, fast-growing trees may be more vulnerable due to higher hydraulic demand and more rapid depletion of stored reserves. Thus, growth–vitality relationships are context-dependent, shaped by tree allometry, drought characteristics and long-term physiological trade-offs.

This trade-off between growth and longevity has been consistently observed across temperate and boreal forests. Dendrochronological and observational studies show that trees with rapid juvenile growth often die younger, whilst slow-growing individuals can attain exceptional ages (Büntgen et al. 2019, Brienen et al. 2020). Several mechanisms may underpin this pattern. Fast-growing trees tend to prioritize biomass production at the expense of structural integrity, defence allocation and carbohydrate storage. For instance, they often form less dense wood with larger vessels, increasing their hydraulic vulnerability during drought conditions (Rosner et al. 2014). Additionally, rapid growth may limit the accumulation of non-structural carbohydrates (NSCs), thereby reducing energy availability for maintaining metabolism, initiating stress-protective responses or repairing damaged tissues during prolonged or repeated drought (Samraoui et al. 2025).

Fast growth may also lead to the earlier onset of size-related constraints, including reduced hydraulic efficiency and greater exposure to mechanical disturbances such as windthrow or snow damage (Di Filippo et al. 2015). In contrast, slower-growing trees tend to invest more in structural defences, NSC reserves and conservative water-use strategies, all of which enhance stress resilience. This supports the view that promoting moderate growth rates may improve forest stability under future climate extremes (Rosner et al. 2014, Büntgen et al. 2019, Brienen et al. 2020, Bose et al. 2024).

Post-drought recovery patterns further illustrate these physiological differences. Tree responses to individual drought events revealed important nuances. After the 1947 and 1976 droughts, Class 3 trees—those classified as poor-vigour after the 2018 drought—recovered relatively quickly, whereas more vigorous trees exhibited delayed recovery (Figure 5a). However, tree responses varied amongst drought events. For instance, the 1976 drought triggered the most pronounced growth decline, likely due to a combination of spring and summer dryness (Figure S5 available as Supplementary Data at *Tree Physiology* Online). In contrast, although the 2003 drought was extremely hot in August, it was preceded by above-average precipitation in May, which may have supported normal earlywood formation and limited immediate growth suppression. Nevertheless, the exceptionally hot and dry late-season conditions appear to have initiated a prolonged growth decline, particularly in Class 3 trees, which never fully recovered. This decline culminated in 2006—a year not initially recognized as extreme—with record July temperatures exceeding those of both 2003 and 2018 at the Mainz climate station (Figure S5 available as Supplementary Data at *Tree Physiology* Online).

These findings support the interpretation that the recent dieback was not solely triggered by the 2018 and 2019 droughts, but rather reflects the cumulative effects of recurring extreme events over the past two decades. Notably, growth has been declining across all vigour classes since 2002—more than 15 years before the most recent mortality episode. This long-term downward trend is consistent with the hypothesis that progressive carbon depletion, or carbon starvation, is a central mechanism of drought-induced tree mortality (Adams et al. 2017, Gessler et al. 2018, McDowell et al. 2022, Bose et al. 2024).

Carbon starvation results from a sustained imbalance between carbon supply and demand—when a tree’s carbon income, derived from photosynthesis and mobilized NSC reserves, chronically falls below the energy required to sustain respiration, growth and defence (Adams et al. 2017, McDowell 2011, McDowell et al. 2022). Prolonged or recurrent droughts exacerbate this imbalance by inducing stomatal closure to minimize water loss, which in turn reduces intercellular CO_2_ concentration and suppresses photosynthetic carbon uptake (Cernusak 2020, Shestakova and Martínez-Sancho 2021). Meanwhile, metabolic demands such as maintenance respiration and osmotic regulation continue, forcing tree reliance on NSC reserves to meet high energy needs during stress conditions (McDowell et al. 2008, Hartmann 2015). If the imbalance persists over several years, vital functions decline and mortality can ensue—a trajectory consistent with carbon starvation, typically preceded by long-term growth decline (Gessler et al. 2018).

### Physiological adjustment revealed by stable isotopes

Building on the observed long-term growth decline and increasing crown damage in Class 3 trees, stable isotope analyses offer valuable insights into physiological mechanisms underlying stress responses and mortality trajectories.

Notably, the vigour classes did not differ markedly in δ^13^C values during individual drought years (Figure 5b, Table 2). All trees showed similarly elevated δ^13^C values, consistent with broadly observed stomatal closure and enhanced iWUE in response to acute drought conditions (Linares and Camarero 2012). Reduced stomatal conductance limits intercellular CO_2_ concentrations in leaves, promoting assimilation of the heavier ^13^C isotope during carboxylation and resulting in higher δ^13^C values in tree rings (Gessler et al. 2018, Cernusak 2020, Pernicová et al. 2024, Treydte et al. 2024). The synchronized δ^13^C values in drought-years across vitality classes suggest a broadly shared short-term stomatal adjustment, supported by strong year-to-year coherence in δ^13^C chronologies (Table 1).

In contrast to the synchronized δ^13^C response, δ^18^O values diverged more distinctly amongst classes during drought events. In particular, trees in Class 3 exhibited stronger δ^18^O enrichment than Class 1 or 2 trees in 2018 (Figure 5c, Table S1 available as Supplementary Data at *Tree Physiology* Online). Several, potentially interacting, mechanisms may explain this pattern. First, increased δ^18^O is generally associated with stomatal closure and enhanced evaporative enrichment of leaf water under dry conditions (Scheidegger et al. 2000, Barbour 2007, Farquhar et al. 2007). In this sense, the δ^18^O response is broadly consistent with the increase in δ^13^C across all classes during drought years. Second, Class 3 trees may have relied more heavily on shallow soil water, which is typically more evaporatively enriched than deeper water reservoirs (Gazis and Feng 2004, Hartl-Meier et al. 2015). This interpretation is consistent with their stronger correlations with soil moisture (Figures S2–S4 available as Supplementary Data at *Tree Physiology* Online) and their systematically higher δ^18^O values after 2003 (Figure 3g). Third, trees with larger crowns or higher historical growth may have experienced greater canopy-level evaporative demand during atmospheric drought (Anderegg et al. 2016, Grossiord et al. 2020), potentially amplifying δ^18^O enrichment. Such effects may be expressed more clearly in δ^18^O than in δ^13^C, because in dominant trees the isotopic effect of stomatal regulation on δ^13^C may be partly offset by simultaneously higher carboxylation rates under favourable light conditions. Lastly, although δ^13^C data do not support consistently open stomata in Class 3 trees, it remains possible that, in severe drought years, some individuals experienced impaired stomatal control—so-called ‘stomatal leakiness’—due to cellular damage or disrupted hormonal and hydraulic signalling pathways (Daszkowska-Golec and Szarejko 2013, López et al. 2022). Such dysfunction could lead to unregulated water loss, accelerated dehydration (Brodribb and Cochard 2009, McDowell et al. 2011) and further δ^18^O enrichment (Colangelo et al. 2017, Timofeeva et al. 2017). Whilst this latter mechanism is less likely given the elevated δ^13^C values, we retain it as a possible contributing factor under extreme stress conditions.

However, over the full 90-year chronology (Figure 3), Class 3 trees consistently exhibit higher δ^13^C values than Classes 1 and 2. This pattern might be interpreted as evidence of chronically reduced stomatal conductance and elevated iWUE. Yet such an explanation alone is insufficient, because these trees also displayed higher radial growth during much of the twentieth century, and their δ^18^O values did not differ systematically from other classes until the most recent drought period.

Long-term offsets in tree-ring δ^13^C can arise not only from drought-driven stomatal regulation, but also from structural and positional differences within the canopy. Tree height, crown exposure and light environment strongly influence δ^13^C by altering photosynthetic demand for CO_2_ and the balance between the CO_2_ assimilation rate and stomatal conductance to gas exchange, independent of water stress (Brienen et al. 2017, Vadeboncoeur et al. 2020). In even-aged stands, dominant individuals typically experience higher irradiance and carbon gain, which may be associated with both wider growth rings and higher δ^13^C values. The persistently higher δ^13^C values in Class 3 trees during their vigorous growth phase therefore likely reflect a combination of canopy position, size-related effects and physiological adjustment, rather than prolonged stomatal limitation alone.

Only in the last two decades, when growth declined and drought frequency increased, does the δ^13^C signal in Class 3 converge with an interpretation of chronic stress. In this late phase, elevated δ^13^C is consistent with sustained increases in iWUE under repeated water limitation, marking a transition from dominance-driven isotopic differences to stress-driven physiological regulation. In this sense, a persistent upward shift in δ^13^C may serve as an early warning of declining hydraulic and carbon balance in vulnerable individuals.

The combined analysis of long-term growth and dual-isotope chronologies reveals contrasting physiological trajectories leading to tree mortality. Whilst δ^13^C integrates stomatal regulation, light-driven photosynthetic demand for CO_2_, and canopy position over decadal timescales, δ^18^O captures episodic increases in evaporative demand and hydraulic strain during extreme drought events. This isotopic complementarity enables clearer distinction between carbon- and water-driven limitations and helps identify individuals approaching critical physiological thresholds.

## Conclusions

Our findings show that long-term physiological imbalances—driven by recurring droughts—underlie the recent dieback of Scots pine at its climatic margin. Carbon starvation and hydraulic failure emerged as interconnected mechanisms, particularly in individuals with historically high growth rates.

Stable isotope signals—especially persistently elevated δ^13^C and episodic δ^18^O enrichment—provided early warning indicators of chronic stress and revealed distinct drought-coping strategies related to rooting depth, water source use and stomatal regulation. Whilst δ^13^C captured long-term iWUE shifts and canopy effects, δ^18^O reflected acute hydraulic strain during extreme droughts, highlighting the diagnostic power of dual-isotope analysis.

Trees with slower growth and more conservative water-use strategies proved more resilient, supporting the idea that traits minimizing water loss and maintaining metabolic stability enhance survival under intensifying climate stress. In essence, slow growers survive—a pattern consistent with broader evidence that rapid growth may increase vulnerability (Büntgen et al. 2019).

## Supplementary Material

Supplementary_materials_tpag059

## Data Availability

The data underlying this article are openly available in ASEP repository at https://doi.org/10.57680/asep.0649201.

## References

[ref1] Adams HD, Zeppel MJB, Anderegg WRL et al. (2017) A multi-species synthesis of physiological mechanisms in drought-induced tree mortality. Nat Ecol Evol 1:1285–1291. 10.1038/s41559-017-0248-x. 29046541

[ref2] Anderegg WRL, Martinez-Vilalta J, Cailleret M et al. (2016) When a tree dies in the forest: scaling climate-driven tree mortality to ecosystem water and carbon fluxes. Ecosystems 19:1133–1147. 10.1007/s10021-016-9982-1.

[ref3] Barbour MM (2007) Stable oxygen isotope composition of plant tissue: a review. Funct Plant Biol 34:83–94. 10.1071/FP06228. 32689335

[ref4] Beguería S, Vicente-Serrano SM (2017) SPEI: Calculation of the standardised precipitation-evapotranspiration index. R package version 1.7. https://CRAN.R-project.org/package=SPEI.

[ref5] Belmecheri S, Lavergne A (2020) Compiled records of atmospheric CO_2_ concentrations and stable carbon isotopes to reconstruct climate and derive plant ecophysiological indices from tree rings. Dendrochronologia 63:125748. 10.1016/j.dendro.2020.125748.

[ref6] Benisiewicz B, Pawełczyk S, Niccoli F, Kabala JP, Battipaglia G (2024) Drought impact on eco-physiological responses and growth performance of healthy and declining *Pinus sylvestris* L. trees growing in a dry area of southern Poland. Forests 15:741. 10.3390/f15050741.

[ref7] Biondi F, Qeadan F (2008) A theory-driven approach to tree-ring standardization: defining the biological trend from expected basal area increment. Tree Ring Res 64:81–96. 10.3959/2008-6.1.

[ref8] Blumenstein K, Bußkamp J, Langer GJ, Langer EJ, Terhonen E (2021) The diplodia tip blight pathogen *Sphaeropsis sapinea* is the most common fungus in scots pines’ mycobiome, irrespective of health status—a case study from Germany. J Fungi 7:607. 10.3390/jof7080607. PMC8396920 34436146

[ref9] Boisvenue C, Running SW (2006) Impacts of climate change on natural forest productivity–evidence since the middle of the 20th century. Glob Chang Biol 12:862–882. 10.1111/j.1365-2486.2006.01134.x.

[ref10] Bose A, Gessler A, Büntgen U, Rigling A (2024) Tamm review: drought-induced scots pine mortality – trends, contributing factors, and mechanisms. For Ecol Manage 561:121873. 10.1016/j.foreco.2024.121873.

[ref11] Brienen RJ, Caldwell L, Duchesne L et al. (2020) Forest carbon sink neutralized by pervasive growth-lifespan trade-offs. Nat Commun 11:4241. 10.1038/s41467-020-17966-z. 32901006 PMC7479146

[ref12] Brienen RJW, Gloor E, Clerici S et al. (2017) Tree height strongly affects estimates of water-use efficiency responses to climate and CO_2_ using isotopes. Nat Commun 8:288. 10.1038/s41467-017-00225-z. 28819277 PMC5561090

[ref13] Brodde L, Adamson K, Julio Camarero J et al. (2019) Diplodia tip blight on its way to the north: drivers of disease emergence in northern Europe. Front Plant Sci 9:1818. 10.3389/fpls.2018.01818. 30687338 PMC6334237

[ref14] Brodribb TJ, Cochard H (2009) Hydraulic failure defines the recovery and point of death in water-stressed conifers. Plant Physiol 149:575–584. 10.1104/pp.108.129783. 19011001 PMC2613726

[ref15] Brun P, Psomas A, Ginzler C, Thuiller W, Zappa M, Zimmermann NE (2020) Large-scale early-wilting response of central European forests to the 2018 extreme drought. Glob Chang Biol 26:7021–7035. 10.1111/gcb.15360. 33091233 PMC7756440

[ref16] Brus DJ, Hengeveld GM, Walvoort DJJ, Goedhart PW, Heidema AH, Nabuurs GJ, Gunia K (2012) Statistical mapping of tree species over Europe. Eur J For Res 131:145–157. 10.1007/s10342-011-0513-5.

[ref17] Bunn AG, Korpela M, Biondi F, Mérian P, Qeadan F, Zang C (2021) dplR: Dendrochronology program library in R. R package version 1.7.2. Available at: https://CRAN.R-project.org/package=dplR (1 June 2026, date last accessed).

[ref18] Büntgen U, Krusic PJ, Piermattei A et al. (2019) Limited capacity of tree growth to mitigate the global greenhouse effect under predicted warming. Nat Commun 10:2171. 10.1038/s41467-019-10174-4. 31092831 PMC6520339

[ref19] Büntgen U, Urban O, Krusic PJ et al. (2021) Recent European drought extremes beyond common era background variability. Nat Geosci 14:190–196. 10.1038/s41561-021-00698-0.

[ref20] Cailleret M, Jansen S, Robert EMR, Desoto L, Aakala T, Antos JA, Martínez-Vilalta J (2017) A synthesis of radial growth patterns preceding tree mortality. Glob Chang Biol 23:1675–1690. 10.1111/gcb.13535. 27759919

[ref21] Camarero JJ (2021) The drought–dieback–death conundrum in trees and forests. Plant Ecol Divers 14:1–12. 10.1080/17550874.2021.1961172.

[ref22] Camarero JJ, Gazol A, Sangüesa-Barreda G, Oliva J, Vicente-Serrano SM (2015) To die or not to die: early warnings of tree dieback in response to a severe drought. J Ecol 103:44–57. 10.1111/1365-2745.12295.

[ref23] Cernusak LA (2020) Gas exchange and water-use efficiency in plant canopies. Plant Biol 22:52–67. 10.1111/plb.12939. 30428160

[ref24] Cherubini P, Battipaglia G, Innes JL (2021) Tree vitality and forest health: can tree-ring stable isotopes be used as indicators? Curr For Rep 7:69–80. 10.1007/s40725-021-00137-8.

[ref25] Colangelo M, Camarero JJ, Battipaglia G, Borghetti M, De Micco V, Gentilesca T, Ripullone F (2017) A multi-proxy assessment of dieback causes in a Mediterranean oak species. Tree Physiol 37:617–631. 10.1093/treephys/tpx002. 28338766

[ref26] Cornes RC, van der Schrier G, van den Besselaar EJM, Jones PD (2018) An ensemble version of the E-OBS temperature and precipitation data sets. J Geophys Res Atmos 123:9391–9409. 10.1029/2017JD028200.

[ref27] Daszkowska-Golec A, Szarejko I (2013) Open or close the gate–stomata action under the control of phytohormones in drought stress conditions. Front Plant Sci 4:138. 10.3389/fpls.2013.00138. 23717320 PMC3652521

[ref28] DeSoto L, Cailleret M, Sterck F et al. (2020) Low growth resilience to drought is related to future mortality risk in trees. Nat Commun 11:545. 10.1038/s41467-020-14300-5. 31992718 PMC6987235

[ref29] Di Filippo A, Pederson N, Baliva M, Brunetti M, Dinella A, Kitamura K et al. (2015) The longevity of broadleaf deciduous trees in northern hemisphere temperate forests: insights from tree-ring series. Front Ecol Evol 3:46. 10.3389/fevo.2015.00046.

[ref30] Dobbertin M (2005) Tree growth as indicator of tree vitality and of tree reaction to environmental stress: a review. Eur J For Res 124:319–333. 10.1007/s10342-005-0085-3.

[ref31] Elling W, Dittmar C, Pfaffelmoser K, Rötzer T (2009) Dendroecological assessment of the complex causes of decline and recovery of the growth of silver fir (*Abies alba* Mill.) in southern Germany. For Ecol Manage 257:1175–1187. 10.1016/j.foreco.2008.10.014.

[ref32] Farquhar GD, Cernusak LA, Barnes B (2007) Heavy water fractionation during transpiration. Plant Physiol 143:11–18. 10.1104/pp.106.093278. 17210909 PMC1761995

[ref33] Fritts HC (1976) Tree rings and climate. Academic Press, London.

[ref34] Fu Z, Ciais P, Bastos A et al. (2020) Sensitivity of gross primary productivity to climatic drivers during the summer drought of 2018 in Europe. Philos Trans R Soc B 375:20190747. 10.1098/rstb.2019.0747. PMC7485099 32892724

[ref35] Gärtner H, Nievergelt D (2010) The core-microtome: a new tool for surface preparation on cores and time series analysis of varying cell parameters. Dendrochronologia 28:85–92. 10.1016/j.dendro.2009.09.002.

[ref36] Gaudinski JB, Dawson TE, Quideau S, Schuur EA, Roden JS, Trumbore SE, Sandquist DR, Oh SW, Wasylishen RE (2005) Comparative analysis of cellulose preparation techniques for use with ^13^C, ^14^C, and ^18^O isotopic measurements. Anal Chem 77:7212–7224. 10.1021/ac050548u. 16285668

[ref37] Gazis C, Feng X (2004) A stable isotope study of soil water: evidence for mixing and preferential flow paths. Geoderma 119:97–111. 10.1016/S0016-7061(03)00243-X.

[ref39] Gessler A, Ferrio JP, Hommel R, Treydte K, Werner RA, Monson RK (2014) Stable isotopes in tree rings: towards a mechanistic understanding of isotope fractionation and mixing processes from the leaves to the wood. Tree Physiol 34:796–818. 10.1093/treephys/tpu040. 24907466

[ref38] Gessler A, Cailleret M, Joseph J et al. (2018) Drought induced tree mortality - a tree-ring isotope based conceptual model to assess mechanisms and predispositions. New Phytol 219:485–490. 10.1111/nph.15154. 29626352

[ref40] Grosjean P, Ibanez F (2018) Pastecs: Package for analysis of space-time ecological series. R package version 1.3.21. https://CRAN.R-project.org/package=pastecs.

[ref1g] Grossiord C, Buckley TN, Cernusak LA, Novick KA, Poulter B, Siegwolf RTW, Sperry JS, McDowell NG (2020) Plant responses to rising vapor pressure deficit. New Phytol 226:1550–1566. 10.1111/nph.16485. 32064613

[ref41] Haberstroh S, Werner C, Grün M, Kreuzwieser J, Seifert T, Schindler D, Christen A (2022) Central European 2018 hot drought shifts scots pine forest to its tipping point. Plant Biol 24:1186–1197. 10.1111/plb.13455. 35869655

[ref42] Harr L, Esper J, Kirchhefer JA, Zhou W, Hartl C (2021) Growth response of *Betula pubescens* Ehrh. To varying disturbance factors in northern Norway. Trees 35:421–431. 10.1007/s00468-020-02043-1.

[ref44] Hartl C, St George S, Konter O, Harr L, Scholz D, Kirchhefer A, Esper J (2019) Warfare dendrochronology: trees witness the deployment of the German battleship Tirpitz in Norway. Anthropocene 27:100212. 10.1016/j.ancene.2019.100212.

[ref43] Hartl C, Schneider L, Riechelmann DFC, Kuhl E, Kochbeck M, Klippel L, Büntgen U, Esper J (2022) The temperature sensitivity along elevational gradients is more stable in maximum latewood density than tree-ring width. Dendrochronologia 73:125958. 10.1016/j.dendro.2022.125958.

[ref47] Hartl-Meier C, Zang C, Dittmar C, Esper J, Göttlein A, Rothe A (2014) Vulnerability of Norway spruce to climate change in mountain forests of the European Alps. Clim Res 60:119–132. 10.3354/cr01226.

[ref46] Hartl-Meier C, Zang C, Büntgen U, Esper J, Rothe A, Göttlein A, Dirnböck T, Treydte K (2015) Uniform climate sensitivity in tree-ring stable isotopes across species and sites in a mid-latitude temperate forest. Tree Physiol 35:4–15. 10.1093/treephys/tpu096. 25466725

[ref45] Hartl-Meier C, Esper J, Liebhold A, Konter O, Rothe A, Büntgen U (2017) Effects of host abundance on larch budmoth outbreaks in the European Alps. Agric For Entomol 19:376–387. 10.1111/afe.12216.

[ref48] Hartmann H (2015) Carbon starvation during drought-induced tree mortality–are we chasing a myth? J Plant Hydraul 2:e005. 10.20870/jph.2015.e005.

[ref49] Holmes RL (1983) Computer-assisted quality control in tree-ring dating and measurement. Tree Ring Bull 43:69–78.

[ref50] Houston Durrant T, de Rigo D, Caudullo G (2016) *Pinus sylvestris* in Europe: distribution, habitat, usage and threats. In: San-Miguel-Ayanz J, de, Rigo D, Caudullo G, Houston Durrant T, Mauri A (eds) European atlas of forest tree species, 2016th edn. Publication Office of the European Union, Luxembourg, p e016b94+.

[ref51] Linares JC, Camarero JJ (2012) From pattern to process: linking intrinsic water-use efficiency to drought-induced forest decline. Glob Chang Biol 18:1000–1015. 10.1111/j.1365-2486.2011.02566.x.

[ref52] López R, Ramírez-Valiente JA, Pita P (2022) How plants cope with heatwaves in a drier environment. Flora 295:152148. 10.1016/j.flora.2022.152148.

[ref53] McDowell N, Pockman WT, Allen CD et al. (2008) Mechanisms of plant survival and mortality during drought: why do some plants survive while others succumb to drought? New Phytol 178:719–739. 10.1111/j.1469-8137.2008.02436.x. 18422905

[ref54] McDowell NG (2011) Mechanisms linking drought, hydraulics, carbon metabolism, and vegetation mortality. Plant Physiol 155:1051–1059. 10.1104/pp.110.170704. 21239620 PMC3046567

[ref55] McDowell NG, Beerling DJ, Breshears DD, Fisher RA, Raffa KF, Stitt M (2011) The interdependence of mechanisms underlying climate-driven vegetation mortality. Trends Ecol Evol 26:523–532. 10.1016/j.tree.2011.06.003. 21802765

[ref56] McDowell NG, Sapes G, Pivovaroff A et al. (2022) Mechanisms of woody-plant mortality under rising drought, CO_2_ and vapour pressure deficit. Nat Rev Earth Environ 3:294–308. 10.1038/s43017-022-00272-1.

[ref57] Meinzer FC, McCulloh KA (2013) Xylem recovery from drought-induced embolism: where is the hydraulic point of no return? Tree Physiol 33:331–334. 10.1093/treephys/tpt022. 23612243

[ref58] MUEEF (Ministry for the Environment, Energy, Food and Forests of Rhineland-Palatinate) (2022) Waldzustandsbericht Rheinland-Pfalz 2022. Mainz, Germany. Available at: https://mkuem.rlp.de/fileadmin/14/Service/Publikationen/Waldzustandsbericht_RLP_2022.pdf (27 January 2026, date last accessed).

[ref59] Muller B, Pantin F, Génard M, Turc O, Freixes S, Piques M, Gibon Y (2011) Water deficits uncouple growth from photosynthesis, increase C content, and modify the relationships between C and growth in sink organs. J Exp Bot 62:1715–1729. 10.1093/jxb/erq438. 21239376

[ref60] Pernicová N, Urban O, Čáslavský J, Kolář T, Rybníček M, Sochová I, Peñuelas J, Bošeľa M, Trnka M (2024) Impacts of elevated CO_2_ levels and temperature on photosynthesis and stomatal closure along an altitudinal gradient are counteracted by the rising atmospheric vapor pressure deficit. Sci Total Environ 921:171173. 10.1016/j.scitotenv.2024.171173. 38401718

[ref61] Peters W, Bastos A, Ciais P, Vermeulen A (2020) A historical, geographical and ecological perspective on the 2018 European summer drought. Philos Trans R Soc B 375:20190505. 10.1098/rstb.2019.0505. PMC7485105 32892723

[ref62] Pretzsch H (2009) Forest dynamics, growth and yield: from measurement to model. Springer Berlin, Heidelberg. 10.1007/978-3-540-88307-4.

[ref1r] R Core Team (2022) R: A language and environment for statistical computing. R Foundation for Statistical Computing, Vienna, Austria.

[ref63] Rosner S, Světlík J, Andreassen K et al. (2014) Wood density as a screening trait for drought sensitivity in Norway spruce. Can J For Res 44:154–161. 10.1139/cjfr-2013-0209.

[ref64] Rybníček M, Kolář T, Ač A, Balek J, Koňasová E, Trnka M, Urban O, Büntgen U (2021) Non-pooled oak (*Quercus* spp.) stable isotopes reveal enhanced climate sensitivity compared with ring widths. Clim Res 83:27–41. 10.3354/cr01632.

[ref65] Samaniego L, Kumar R, Zink M (2013) Implications of parameter uncertainty on soil moisture drought analysis in Germany. J Hydrometeorol 14:47–68. 10.1175/JHM-D-12-075.1.

[ref66] Samraoui KR, Klimeš A, Jandová V et al. (2025) Trade-offs between growth, longevity, and storage carbohydrates in herbs and shrubs: evidence for active carbon allocation strategies. Plant Cell Environ 48:4505–4517. 10.1111/pce.15444. 40016866 PMC12050394

[ref67] Scheidegger Y, Saurer M, Bahn M, Siegwolf R (2000) Linking stable oxygen and carbon isotopes with stomatal and photosynthetic capacity: a conceptual model. Oecologia 125:350–357. 10.1007/s004420000466. 28547329

[ref68] Schuldt B, Buras A, Arend M et al. (2020) A first assessment of the impact of the extreme 2018 summer drought on central European forests. Basic Appl Ecol 45:86–103. 10.1016/j.baae.2020.04.003.

[ref69] Schütt P, Cowling EB (1985) Waldsterben, a general decline of forests in Central Europe: symptoms, development, and possible causes. Plant Dis 69:548–558.

[ref70] Seidl R, Schelhaas M-J, Rammer W, Verkerk PJ (2014) Increasing forest disturbances in Europe and their impact on carbon storage. Nat Clim Change 4:806–810. 10.1038/nclimate2318. PMC4340567 25737744

[ref71] Seidling W, Ziche D, Beck W (2012) Climate responses and interrelations of stem increment and crown transparency in Norway spruce, scots pine, and common beech. For Ecol Manage 284:196–204. 10.1016/j.foreco.2012.07.015.

[ref73] Senf C, Seidl R (2021) Persistent impacts of the 2018 drought on forest disturbance regimes in Europe. Biogeosciences 18:5223–5230. 10.5194/bg-18-5223-2021.

[ref72] Senf C, Buras A, Zang CS, Rammig A, Seidl R (2020) Excess forest mortality is consistently linked to drought across Europe. Nat Commun 11:6200. 10.1038/s41467-020-19924-1. 33273460 PMC7713373

[ref74] Shestakova TA, Martínez-Sancho E (2021) Stories hidden in tree rings: a review on the application of stable carbon isotopes to dendrosciences. Dendrochronologia 65:125789. 10.1016/j.dendro.2020.125789.

[ref75] Suzuki N, Rivero RM, Shulaev V, Blumwald E, Mittler R (2014) Abiotic and biotic stress combinations. New Phytol 203:32–43. 10.1111/nph.12797. 24720847

[ref76] Tallieu C, Badeau V, Allard D, Nageleisen LM, Bréda N (2020) Year-to-year crown condition poorly contributes to ring width variations of beech trees in French ICP level I network. For Ecol Manage 465:118071. 10.1016/j.foreco.2020.118071.

[ref77] Teshome DT, Zharare GE, Naidoo S (2020) The threat of the combined effect of biotic and abiotic stress factors in forestry under a changing climate. Front Plant Sci 11:601009. 10.3389/fpls.2020.601009. 33329666 PMC7733969

[ref78] Tew ER, Ambrose-Oji B, Beatty M et al. (2024) A horizon scan of issues affecting UK forest management within 50 years. For Int J For Res 97:349–362. 10.1093/forestry/cpad047.

[ref79] Timofeeva G, Treydte K, Bugmann H, Rigling A, Schaub M, Siegwolf R, Saurer M (2017) Long-term effects of drought on tree-ring growth and carbon isotope variability in scots pine in a dry environment. Tree Physiol 37:1028–1041. 10.1093/treephys/tpx041. 28444356

[ref80] Treydte K, Boda S, Graf Pannatier E et al. (2014) Seasonal transfer of oxygen isotopes from precipitation and soil to the tree ring: source water versus needle water enrichment. New Phytol 202:772–783. 10.1111/nph.12741. 24602089

[ref81] Treydte K, Liu L, Padrón RS et al. (2024) Recent human-induced atmospheric drying across Europe unprecedented in the last 400 years. Nat Geosci 17:58–65. 10.1038/s41561-023-01335-8.

[ref82] Trumbore S, Brando P, Hartmann H (2015) Forest health and global change. Science 349:814–818. 10.1126/science.aac6759. 26293952

[ref83] Ulrich B, Pankrath J (1983) Effects of accumulation of air pollutants in forest ecosystems: Proceedings of a workshop held at Göttingen, West Germany, May 16–18, 1982. Springer Science & Business Media, Dordrecht. 10.1007/978-94-009-6983-4.

[ref84] Urban O, Ač A, Kolář T, Rybníček M, Pernicová N, Koňasová E, Trnka M, Büntgen U (2021) The dendroclimatic value of oak stable isotopes. Dendrochronologia 65:125804. 10.1016/j.dendro.2020.125804.

[ref85] Vadeboncoeur MA, Jennings KA, Ouimette AP, Asbjornsen H (2020) Correcting tree-ring δ^13^C time series for tree-size effects in eight temperate tree species. Tree Physiol 40:333–349. 10.1093/treephys/tpz138. 31976526

[ref86] Vicente-Serrano SM, Beguería S, López-Moreno JI (2010) A multiscalar drought index sensitive to global warming: the standardized precipitation evapotranspiration index. J Clim 23:1696–1718. 10.1175/2009JCLI2909.1.

[ref87] Wellbrock N, Eickenscheidt N, Hilbrig L et al. (2018) Leitfaden und Dokumentation zur Waldzustandserhebung in Deutschland. Thünen Working Paper, No. 84. Johann Heinrich von Thünen-Institut, Braunschweig, Germany. 10.3220/WP1513589598000.

[ref88] Zang C, Biondi F (2015) Treeclim: an R package for the numerical calibration of proxy-climate relationships. Ecography 38:431–436. 10.1111/ecog.01335.

