# Supplementary material for: Tree-ring stable isotopes and growth trajectories reveal early warning signals of drought-induced Scots pine mortality
Source: Tree Physiol. 2026 Apr 30;46(6):tpag059. doi: 10.1093/treephys/tpag059 (PMC13253043; doi:10.1093/treephys/tpag059)
Supplement: Supplementary_materials_tpag059 [file supplementary_materials_tpag059.zip › Supplementary_materials_tpag059_Figures.docx]

**Supplementary Material**

O. Urban *et al.*: Tree-ring stable isotopes and growth trajectories reveal early warning signals of drought-induced Scots pine mortality

**Figure S1.** Interannual variability in canopy greenness and red-edge chlorophyll-proxy indices.

**Figure S2.** Monthly response of BAI to environmental variables.

**Figure S3.** Monthly response of δ^13^C to environmental variables.

**Figure S4.** Monthly response of δ^18^O to environmental variables.

**Figure S5.** Climatic conditions during specific years (lines) with respect to the 1961-1990 reference period (area signatures) based on DWD climate data from Mainz.

**Figure S1.** Interannual variability in canopy greenness and red-edge chlorophyll-proxy indices for a 2-ha region of interest (ROI) centred at 50°00'44.0"N, 8°11'23.7"E in the Lenneberg Forest (Mainz, western Germany). Filled circles indicate Landsat-derived NDVI (2000–2025), while open circles represent a combined series of red-edge NDRE values: Landsat-based NDREeq (NDRE-equivalent) estimates for 2000–2016 and Sentinel-2 NDRE measurements for 2017–2025. Each point reflects the mean of summer (June–August) per-pixel median composites across all valid cloud-free pixels within the ROI; error bars indicate ±1 SD (spatial heterogeneity). Vertical red lines mark the extreme drought years 2003 and 2018, which coincide with marked drops in NDVI and NDRE. NDVI = (NIR − red)/(NIR + red), derived from Landsat sensors using standard near-infrared and red bands. Sentinel-2 NDRE = (NIR − red-edge)/(NIR + red-edge), calculated from bands B8 (842 nm) and B5 (705 nm). Due to the absence of red-edge bands in Landsat, NDREeq values were calculated via linear regression between Sentinel-2 NDRE and overlapping Landsat NDVI observations from 2017–2025 (NDREeq = 0.0514 + 0.6814 × NDVI; R^2^ = 0.61, n = 9). Data processing was performed in Google Earth Engine (GEE); see Methods for details.

**Remote-sensing data retrieval and index calculation.** Remote-sensing processing was performed in Google Earth Engine (GEE). The ROI was defined as a circular buffer with an area of 2 ha around the centre coordinates given above. Landsat data were obtained from Collection 2, Tier 1, Level 2 surface reflectance products for Landsat 5 TM (LANDSAT/LT05/C02/T1_L2), Landsat 7 ETM+ (LANDSAT/LE07/C02/T1_L2), Landsat 8 OLI (LANDSAT/LC08/C02/T1_L2) and Landsat 9 OLI-2 (LANDSAT/LC09/C02/T1_L2). Sentinel-2 data were obtained from the harmonized surface reflectance product (COPERNICUS/S2_SR_HARMONIZED). For each sensor and year, all scenes intersecting the ROI in June–August were collected. Clouds, cloud shadows, cirrus and snow/ice were masked using the QA_PIXEL bitmask for Landsat (cloud shadow, snow, cloud, cirrus) and the Scene Classification Layer for Sentinel-2 (SCL classes 3, 8–11). Landsat reflectance scaling followed the Collection 2 Level 2 definition (reflectance = DN × 0.0000275 − 0.2). Annual summer composites were calculated using the per-pixel median of all valid observations within the seasonal window. ROI-level means and standard deviations were computed from the composite images at the native spatial resolution (30 m for Landsat; 10 m for Sentinel-2). Years with no valid observations after masking were treated as missing. The combined NDRE series in this figure uses Landsat-derived NDREeq for 2000–2016 and Sentinel-2 NDRE for 2017–2025.


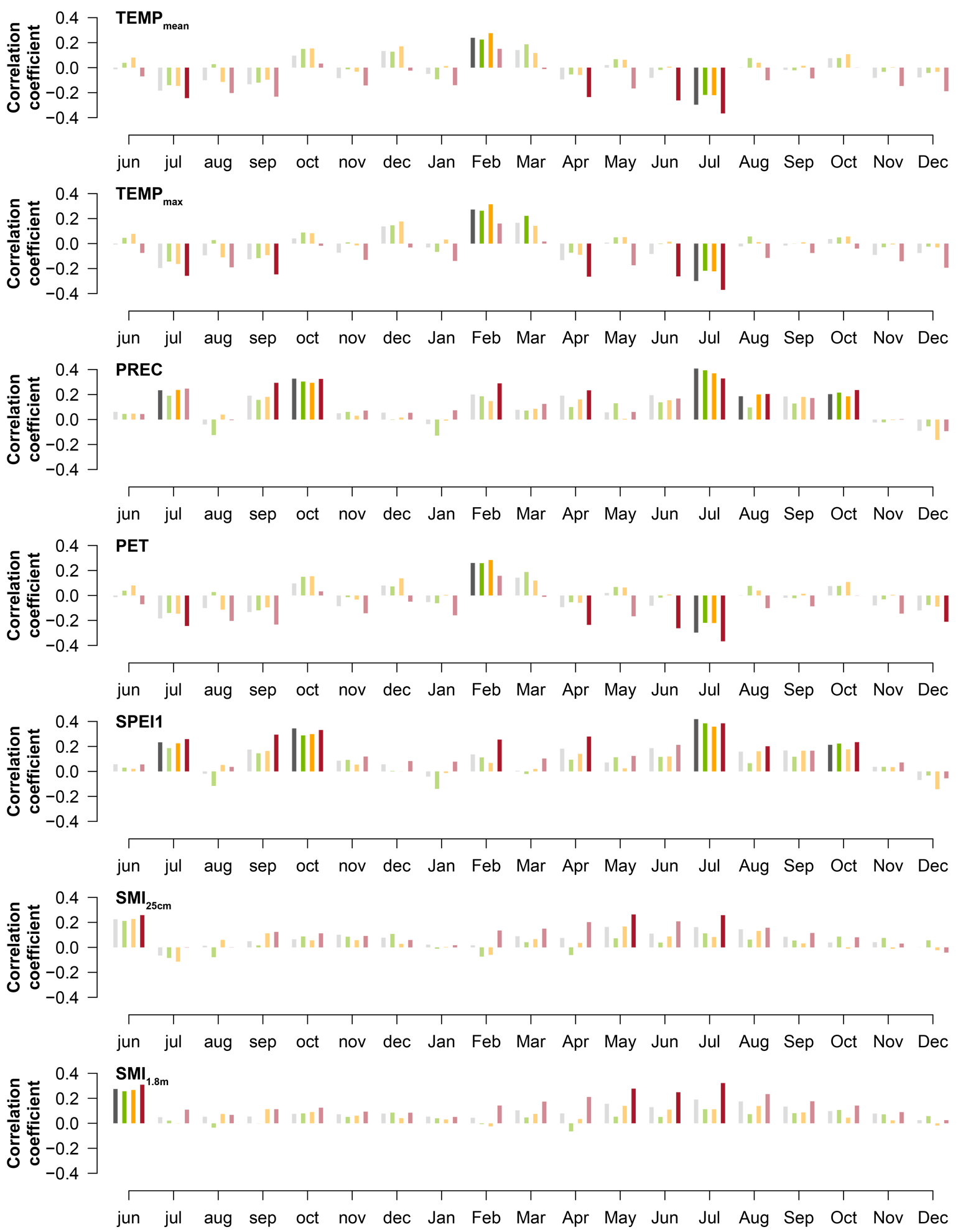


**Figure S2.** Monthly response of basal area increment (BAI) to environmental variables. Values of correlation coefficients between monthly environmental variables and BAI for the period 1930–2019. Colours denote vitality classes: dark green = all trees combined, light green = Class 1 (vigorous), yellow = Class 2 (intermediate), red = Class 3 (poor vigour). Transparent bars indicate non-significant correlations (p ≥ 0.05). Climate variables include monthly temperature, precipitation, potential evapotranspiration (PET), and soil moisture index (SMI). See Methods for data sources and correlation procedures.


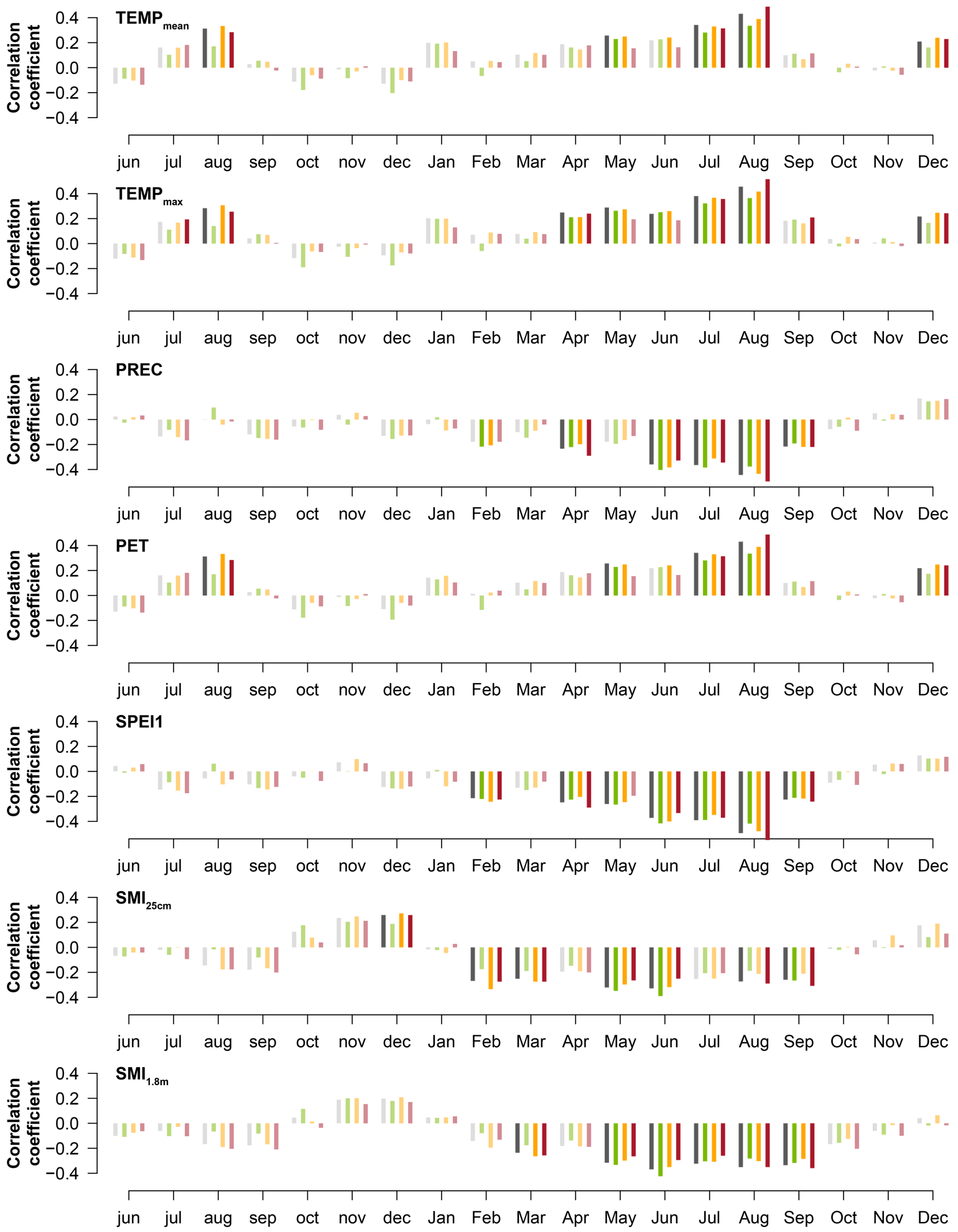


**Figure S3.** Monthly response of δ^13^C to environmental variables. Correlation coefficients between monthly climate parameters and tree-ring δ^13^C values over the period 1930–2019. Vitality classes are colour-coded as in Fig. S2. Statistically non-significant correlations are shown as transparent bars. δ^13^C values were corrected for atmospheric changes (Suess effect); see Methods for analytical and statistical details.


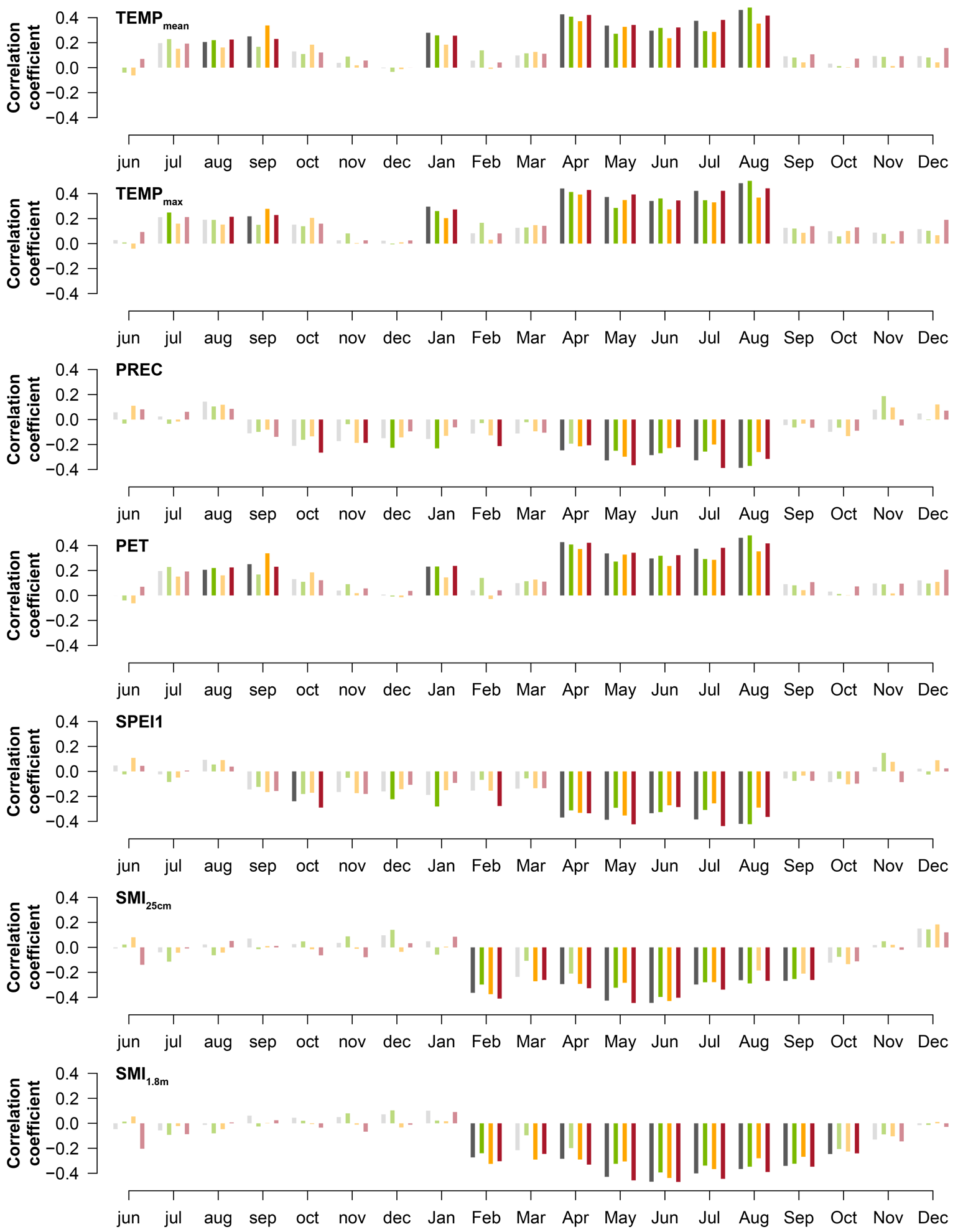


**Figure S4.** Monthly response of δ^18^O to environmental variables. Values of correlation coefficients between monthly climate parameters and δ^18^O values in tree-ring cellulose from 1930–2019. Colours and significance indications follow the conventions used in Figs. S2 and S3. See Methods for isotope analysis and climate data processing.


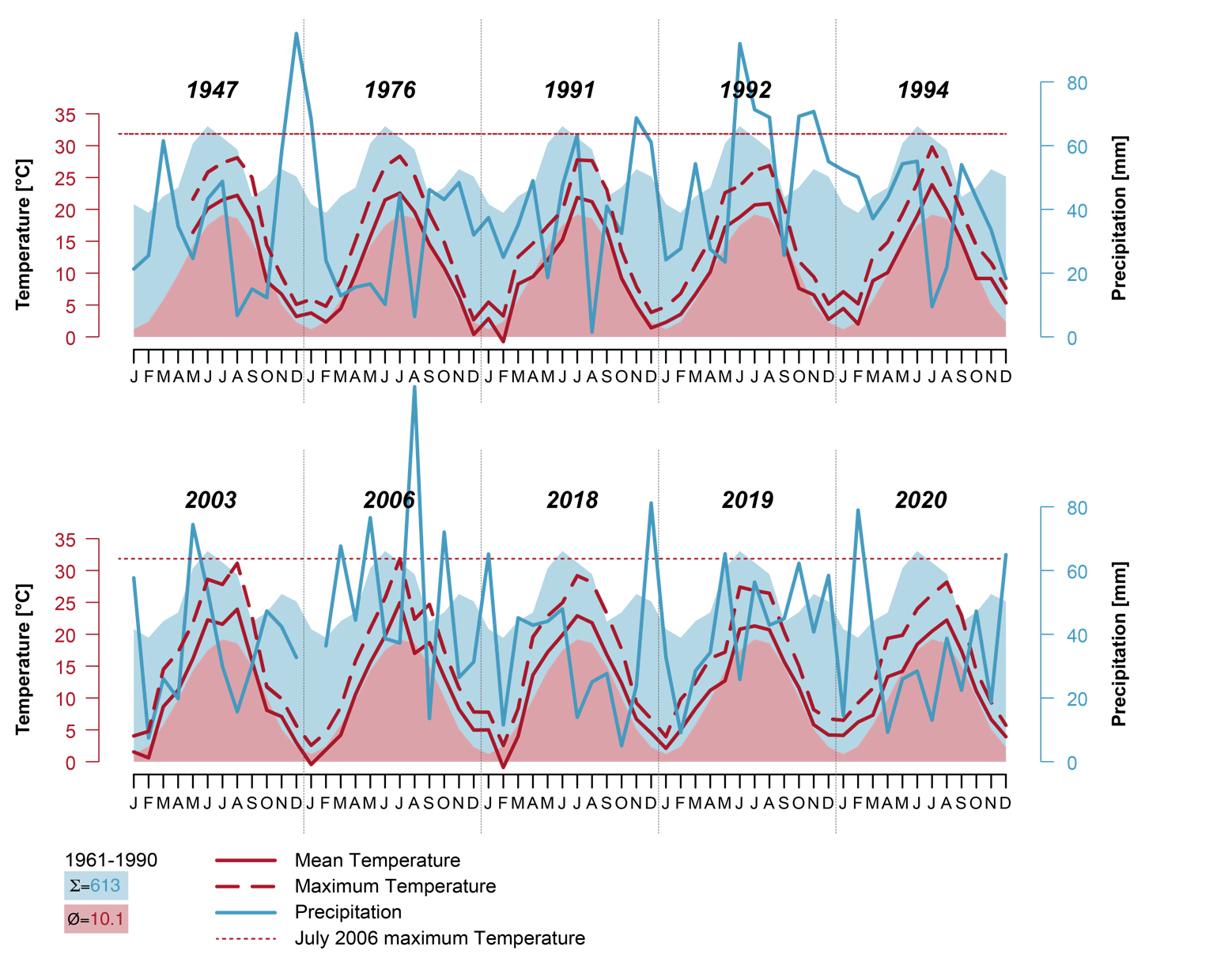


**Figure S5.** Climatic conditions during specific years (lines) with respect to the 1961–1990 reference period (area signatures) based on DWD climate data from Mainz.
